# Efficacy of Xenogeneic Collagen Matrices in Augmenting Peri‐Implant Soft Tissue: A Systematic Review and Meta‐Analysis

**DOI:** 10.1002/cre2.937

**Published:** 2024-08-05

**Authors:** Shashi Dadlani, Betsy Joseph, Sukumaran Anil

**Affiliations:** ^1^ Section of Periodontology, Faculty of Medicine and Health Sciences, University Clinic of Dentistry University of Oviedo Oviedo Spain; ^2^ Department of Periodontics Saveetha Institute of Medical and Technical Sciences, Saveetha Dental College and Hospitals Chennai India; ^3^ Department of Oral and Maxillofacial Diseases Helsinki University and University Hospital Helsinki Finland; ^4^ Department of Dentistry, Oral Health Institute, Hamad Medical Corporation University Doha Doha Qatar

**Keywords:** dental implants, peri‐implant soft tissue, soft tissue augmentation, xenogeneic collagen matrix

## Abstract

**Background:**

Xenogenic collagen matrices (XCMs) are gaining popularity for soft tissue augmentation in dental implants; yet, gaps exist in our understanding of their comparative effectiveness.

**Objective:**

This systematic review and meta‐analysis focuses on studies that utilize soft tissue augmentation techniques for dental implants to improve keratinized mucosa width (KMW), soft tissue thickness (STT), and soft tissue volume (STV). We compared porcine collagen matrices with autogenous grafts when no bone grafts were utilized.

**Materials and Methods:**

We searched databases such as PubMed, Scopus, and the Cochrane Central Register of Controlled Trials for randomized controlled trials and controlled clinical trials published between January 2013 and July 2023 that assessed the efficacy of XCM in peri‐implant soft tissue augmentation. The primary outcome included KMW changes while the secondary outcome was STT/STV changes. Statistical analyses were conducted using a random‐ or fixed‐effects model, and heterogeneity was assessed using *I* ^2^ statistics.

**Results:**

Nine studies were included in the qualitative analysis, and six were included in the meta‐analysis. No significant intergroup differences were observed (*p* > 0.05), but a significant difference was observed in favor of KMW ≥ 2 mm. Heterogeneity among the studies varied at the 6‐ and 12‐month follow‐ups, with *I* ^2^ values of 78% and 0%, respectively. The pooled mean difference between the XCM and autograft groups was −0.96 (−1.71 to −0.21), which shows that there was a larger increase in KMW in the autograft group compared with the XCM group (*p* < 0.05).

**Conclusions:**

Collagen matrices are less effective than autogenous grafts at increasing keratinized tissue and STT/STV, but the two techniques yield comparable aesthetic outcomes. Additional studies are necessary to better guide clinical practice and improve patient outcomes.

## 1 Introduction

Dental implants have revolutionized restorative dentistry, offering a stable and aesthetically pleasing solution to edentulism. Following tooth extraction, alveolar bone resorption becomes pronounced, especially in the aesthetic zone, often leading to adverse patient experiences (Cardaropoli, Araújo, and Lindhe 2003; Araújo and Lindhe 2005; Chen and Buser 2009). Research indicates that post‐extraction bone remodeling can reduce alveolar ridge width by up to 50% within a year (Schropp et al. 2003). Concurrently, post‐extraction soft tissue alterations can significantly influence implant‐supported prostheses' aesthetic and functional outcomes (Chappuis et al. 2015; Chappuis, Araújo, and Buser 2017). Beyond osseointegration of the implant into the alveolar bone, harmonious integration with surrounding soft tissues is equally paramount.

Maintaining sufficient soft tissue volume (STV) and contour is crucial for minor buccal dehiscences around dental implants. Adequate soft tissue thickness (STT) is vital in preventing crestal bone loss, sealing the implant area to prevent bacterial ingress into the gingival sulcus, and achieving a desirable aesthetic appearance (Abou‐Arraj et al. 2020; De Angelis et al. 2021; Thoma et al. 2021). Studies have established that a minimum of 2 mm width of keratinized mucosa surrounding implants is requisite to minimize patient discomfort during brushing, reduce peri‐implant tissue inflammation, and regulate plaque accumulation (Gharpure et al. 2021; Kabir, Stiesch, and Grischke 2021; Shimomoto et al. 2021). When the keratinized mucosa width (KMW) is less than 2 mm, as concurred in the Consensus Report by Group 1 of the DGI/SEPA/Osteology Workshop, soft tissue augmentation is definitively recommended, particularly in cases of peri‐implant inflammation, pain, or brushing challenges (Giannobile, Jung, and Schwarz 2018).

Soft tissue augmentation, aimed at improving tissue quantity and quality around dental implants, plays a significant role in implant‐supported prostheses' aesthetic and functional success (Fickl et al. 2021). Connective tissue grafts (CTGs) are recognized as a practical solution and are often hailed as the gold standard (Puzio et al. 2020; Vallecillo et al. 2021). However, CTGs are associated with disadvantages, including intra‐ and postoperative hemorrhage, potential damage to the palatine artery, graft necrosis, limited graft availability, and heightened patient discomfort (Dadlani 2021; Ripoll et al. 2021; Schinini et al. 2021). These challenges have propelled the search for alternative solutions, such as collagen matrices. While various materials have been employed, xenogeneic collagen matrices (XCMs) have emerged as a prominent choice due to their availability, volume stability over time, biocompatibility, ease of use, and promising clinical outcomes (Patil and Masters 2020). Though collagen matrices have demonstrated efficacy in increasing keratinized mucosa around implants, as substantiated by several randomized clinical trials (RCTs) (Lorenzo et al. 2012; Cairo et al. 2017; Qiu et al. 2023), there is a paucity of evidence supporting their superiority over CTGs in amplifying STT or STV (Lorenzo et al. 2012; Schmitt et al. 2021; Hammerle et al. 2023).

While XCMs are increasingly adopted for soft tissue augmentation around dental implants, a comprehensive understanding of their differential efficacies remains limited. Preliminary investigations suggest variations in clinical outcomes, patient satisfaction, and potential complications among different xenogeneic matrices (De Angelis et al. 2021; Fickl et al. 2021). This systematic review and meta‐analysis aim to meticulously examine the existing evidence, explicitly focusing on studies employing soft tissue augmentation techniques to enhance peri‐implant KMW, STT, and STV. Other alternatives, such as allogenic matrices, have raised ethical concerns; hence, special emphasis is laid on using porcine collagen matrices, a modality gaining momentum in contemporary peri‐implant surgical procedures.

## 2 Methodology

The research question guiding this systematic review was articulated as follows: “In patients requiring soft tissue augmentation around dental implants, to what extent are XCMs efficacious in enhancing the KMW, STT, and STV in comparison to autogenous grafts in the absence of bone grafts?”

### 2.1 Protocol and Registration

The formulation of the research problem, articulation of the focused question, and delineation of the inclusion and exclusion criteria emerged after an initial review of extant literature. After that, a formal protocol was meticulously developed and registered with the International Prospective Register of Systematic Reviews (PROSPERO) before the systematic review process (CRD42023455643).

### 2.2 Eligibility Criteria

The PICO (Population, Intervention, Comparisons, Outcomes) model was employed to formulate the focus question precisely. The question was structured thus: In patients necessitating soft tissue augmentation in proximity to dental implants (P), to what extent are XCMs (I) efficacious in augmenting the width of keratinized mucosa and the STV (O), in comparison to autogenous grafts, in the absence of bone graft utilization (C)?

The eligibility criteria for the studies to be included are categorized as follows: (1) Clinical investigations conducted on human subjects, specifically RCTs and controlled clinical trials (CCTs); (2) studies featuring a follow‐up period exceeding 6 months; (3) studies comprising more than 10 individual cases; (4) prospective cohort studies; and (5) investigations evaluating the impact of XCMs on either the enhancement of KMW or the amplification of STV or STT around dental implants, relative to other autogenous grafts, in settings where bone grafts are not employed. Publications appearing in the peer‐reviewed literature between 2013 and 2023 were selected.

Conversely, studies meeting the following criteria were systematically excluded: case reports, studies on animal subjects, in vitro investigations, literature reviews, editorials, consensus papers, articles using bone grafts, allografts, XCMs not from porcine origin, and articles not published in the English language

### 2.3 Information Sources and Search Strategies

This systematic review was conducted and reported following the PRISMA (Preferred Reporting Items for Systematic Reviews and Meta‐Analyses) guidelines (Liberati et al. 2009). Studies that conformed to the PICO criteria, as mentioned earlier, and were published between 2013 and 2023 were deemed eligible for inclusion. A comprehensive search was executed across major academic databases, including but not limited to PubMed, Scopus, and the Cochrane Central Register of Controlled Trials to identify studies that satisfy the eligibility criteria.

The search strategy was predicated upon three key concepts related to the research question: XCM, soft tissue augmentation, and dental implants. Utilizing a combination of MeSH (Medical Subject Headings) terms, keywords, and accessible terms related to these concepts, the literature was systematically searched to identify pertinent publications from 2013 to 2023. To enhance the database search, we also used other methods like checking the references in relevant studies and manually searching to find additional publications that might be missed by the database.

### 2.4 Study Selection

In the initial phase, articles were systematically identified through a database search, utilizing keywords related to the research question's primary concepts. After this, reviewers S.D. and B.J. scrutinized the articles' titles and abstracts, making preliminary selections based on the predetermined inclusion and exclusion criteria. Consequently, duplicate articles were removed from the data set using reference management software, EndNote X9 (Thomson Reuters, Philadelphia, PA, USA).

After that, the full texts of the remaining articles were downloaded and examined by the reviewers S.D. and B.J. When disagreements arose concerning the eligibility of specific articles, a third reviewer, S.A., was brought in to help resolve the issue through discussion. This collaborative process led to a final agreement on which articles to include or exclude.

### 2.5 Data Extraction and Data Items

Before the formal study selection, a pilot phase was executed to refine the inclusion criteria and validate the inter‐rater reliability, thereby ensuring that multiple reviewers could apply the criteria with a consistent interpretative framework. After selecting eligible studies, data were systematically extracted and organized into tabular forms using Microsoft Excel spreadsheets.

Two reviewers, S.D. and B.J., conducted independent data extraction, focusing on the following variables: author(s), publication year, country of origin, study design and type, time of register, sample size, specific treatments administered, duration of follow‐up, and baseline and terminal measurements of KMW, STT, and STV, as well as the study's primary conclusions. This process followed the same collaborative approach used in the study selection phase. Both reviewers independently verified the data to reduce errors or biases. When disagreements arose, they were resolved through discussions with a third reviewer, S.A.

### 2.6 Risk of Bias in Individual Studies

Various critical appraisal tools were used depending on the study design to evaluate the risk of bias in individual studies. The Cochrane Collaboration's risk of bias tool (Higgins et al. 2011) was utilized for randomized trials. This tool systematically assesses multiple dimensions of bias, including but not limited to selection bias (via random sequence generation and allocation concealment), performance bias (through blinding of participants and personnel), detection bias (via blinding of outcome assessment), attrition bias (through analysis of incomplete outcome data), reporting bias (via examination of selective reporting), and a composite measure of the overall risk of bias.

The ROBINS‐I (Risk Of Bias In Non‐randomized Studies‐of Interventions) tool (Sterne et al. 2016) was implemented for nonrandomized studies. This tool provides a robust framework for evaluating multiple bias components, including bias due to confounding, bias in the selection of participants into the study, bias in the classification of interventions, bias attributable to deviations from intended interventions, bias due to missing data, bias in the measurement of outcomes, bias in the selection of the reported result, and a culminating evaluation of the overall risk of bias.

### 2.7 Meta‐Analysis

In synthesizing the data for meta‐analytic evaluation, outcome variables were extracted from individual studies and subsequently processed using REVMAN software. The mean differences were calculated for continuous outcomes, accompanied by a 95% confidence interval (CI), to provide a succinct summary of each study's results. The meta‐analysis employed two analytical paradigms: the fixed‐effects model and the random‐effects model. The selection between these models was dictated by the degree of statistical heterogeneity among the studies.

Conversely, a fixed‐effects meta‐analysis was executed when the statistical heterogeneity was minimal, as indicated by an *I* ^2^ value of less than 60% and a *p* value of less than 0.05. Given the limited number of studies incorporated into the meta‐analysis (fewer than 10), standard publication bias diagnostics such as funnel plots and Egger tests were considered inappropriate and, therefore, were not conducted. Statistical significance was established at a *p* value threshold of less than 0.05. Lastly, a sensitivity analysis was carried out, purposefully omitting studies with low methodological quality or unclear biases. This step was undertaken to scrutinize the robustness of the final effect estimates.

## 3 Results

### 3.1 Study Selection

**Figure 1:**
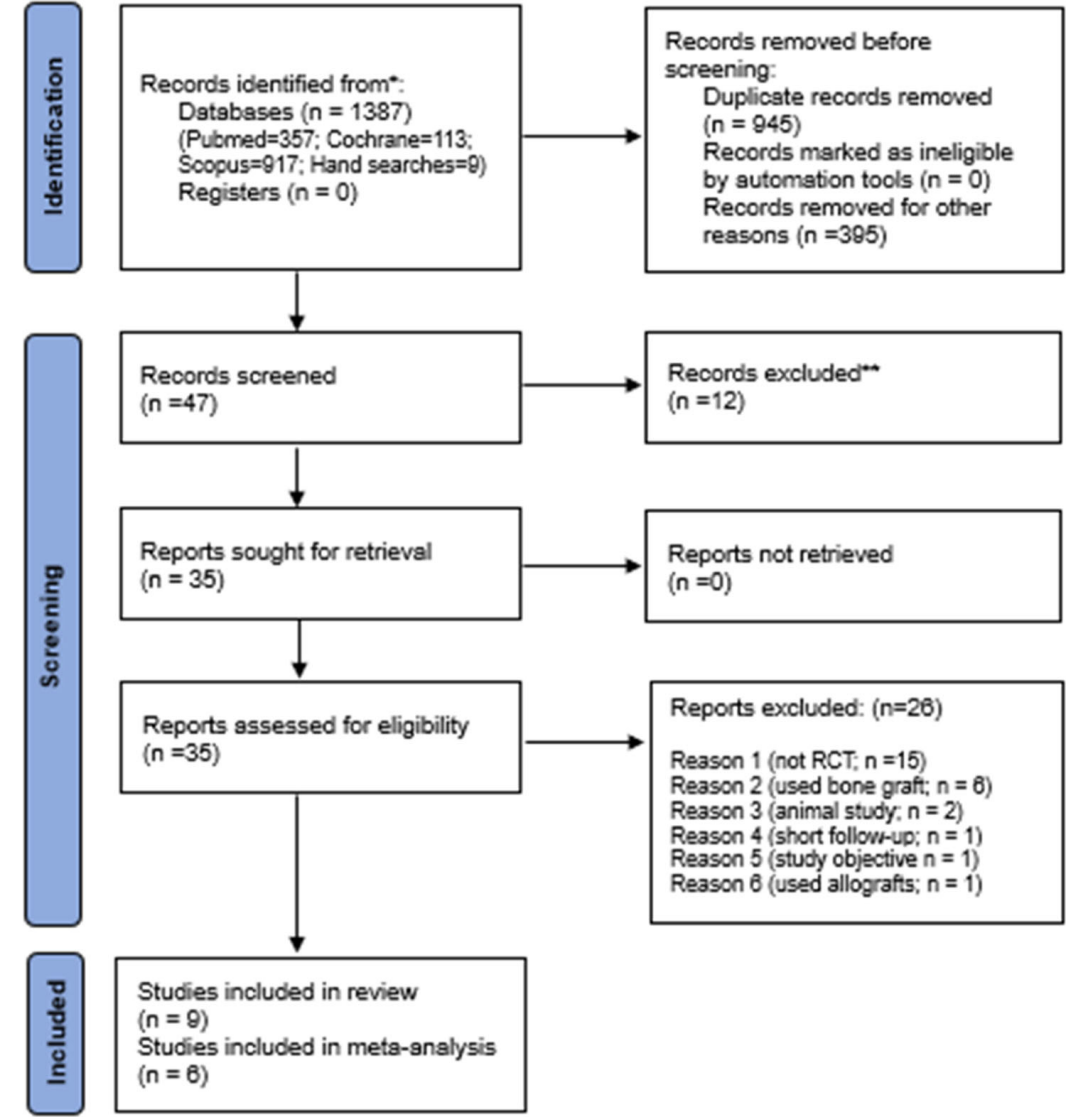
PRISMA flowchart for the review.

One thousand three hundred and eighty‐seven studies were identified by searching various databases. Forty‐seven studies were identified after duplicate removal, and 12 were removed during screening for title and abstract. The remaining 35 studies were subjected to full‐text reading and assessed for eligibility. Finally, nine studies were included in the review. Out of these, six studies were included in the meta‐analysis. Three studies were not included as some data were unavailable (Figure 1).

### 3.2 Study Characteristics

For each included study, the following characteristics were extracted: author, year, country of origin, study design, treatment groups, sample size of each treatment group, follow‐up duration, baseline and final values of KMW and STT/STV, and key findings of the study (Table 1).

**Table 1:** Basic characteristics and key findings of the included studies.

Author (year), region	Study design	Design type	Time of register	Treatment groups; sample size (test/control)	Follow‐up	Baseline keratinized mucosa width in mm (mean ± SD)	Baseline soft tissue thickness in mm (mean ± SD)/volume (mm^3^)	Final keratinized mucosa width mm (mean ± SD)	Final soft tissue volume in mm (mean ± SD)/volume (mm^3^)	Final outcome in mm (mean ± SD)	Conclusion
Thoma et al. (2023), Switzerland	RCT	Parallel	Retrospective	VCMX/SCTG; 20 (10/10) Fibro‐Gide	5 years	KT: 2.4 ± 0.8	STT: 3.2 ± 0.8	KT: 3.1 ± 0.9	STT: 3.4 ± 1.2	KT: 0.6 ± 0.5 STT: 0.3 ± 1.1	VCMX and SCTG showed similar results in soft tissue augmentation, favorable aesthetics, and clinically negligible contour changes at 5 years after loading.
Qiu et al. (2023), China	RCT	Parallel	Prospective	XCM + APF/FGG + APF; 30 (15/15) Mucograft	6 months	KMW: 1.13 ± 0.40	KMT: 1.14 ± 0.30	KMW: 3.28 ± 0.96	KMT: 0.95 ± 0.29	KMW: +2.15 ± 1.12 KMT:−0.19 ± 0.12	XCM plus APF was similar to FGG plus APF in augmenting KMW but exhibited greater shrinkage. XCM plus APF was less effective in augmenting KMT. Aesthetic outcomes were superior with XCM plus APF compared to FGG plus APF.
Ramanauskaite et al. (2023), Germany	RCT	Parallel	Not mentioned	Porcine derived CM/FGG; 32 (15/17) Mucograft	6 months	KT: 0.91 ± 0.76	NR	KT: 2.36 ± 1.11	NR	KT: 1.45 ± 1.13	Three‐dimensional thickness changes were observed in CM and FGG between 1 and 6 months. While FGG resulted in a wider KT band, CM significantly reduced both surgical time and patients' intake of analgesics.
Solonko et al. (2022), Spain	RCT	Parallel	Prospective	APF + CM/APF + FGG; 49 (23/26) Mucograft	1 year	KMW: 0.4 ± 0.5	NR	KMW: 2.0 ± 1.2	NR	KMW: 1.6 ± 1.2	KMW increased significantly with APF + FGG compared to APF + CM; however, CM was more favorably perceived by patients in terms of pain experience and analgesic consumption, despite comparable surgical durations.
Cosyn et al. (2022), Belgium	RCT	Parallel	Prospective	CMX/CTG; 60 (30/30) Fibro‐Gide	1 year	Increase in BSP: 1.90 mm (98.3% CI: 1.58–2.23)	Volume gain: 50.93 mm^3^	Increase in BSP of 0.57 mm (98.3% CI: 0.34–0.79)	Volume gain: 16.92 mm^3^	Final increase in BSP of 0.57 mm (98.3% CI: 0.34–0.79)	CTG continues to serve as the gold standard for increasing soft tissue thickness at implant sites. Clinicians are advised to carefully weigh the benefits of CMX against the considerable risk of graft resorption.
Schmitt et al. (2021), Germany	CCT	Parallel	Not mentioned	Porcine CM/SCTG; 14 (7/7) Mucoderm	6 months	NR	NR	NR	Volume increase: 19.56 ± 8.95 mm^3^	Mean soft tissue thickness increase in the buccal contour: 0.30 ± 0.16 mm	The early healing phase is characterized by a significant reduction in soft tissue volume. The SCTG demonstrates negligible superiority compared to CM.
Huang et al. (2021), China	RCT	Parallel	Retrospective	XCM/FGG; 26 (13/13) Mucograft	6 months	KMW: 0.9 ± 0.6	KMT: 1.1 ± 0.4	KMW: 2.8 ± 1.0	KMT: 1.2 ± 0.3	KMW: 1.8 ± 1.0 KMT: 0.1 ± 0.5	A more substantial increase in KMW and thicker mucosa was observed with FGG compared to XCM. Both modalities effectively increased KMW, maintained peri‐implant health, and yielded comparable aesthetic outcomes. XCM was associated with reduced operation time.
Thoma et al. (2020), Switzerland	RCT	Parallel	Retrospective	VCMX/SCTG; 20 (10/10) Fibro‐Gide	3 years	KT:2.4 ± 0.8	STT: 3.2 ± 0.8	KT: 2.5 ± 1.4	STT: 3.6 ± 1.5	STT: 0.44 ± 1.1	Both modalities exhibited minimal alterations in peri‐implant tissue contour and in soft tissue thickness, which experienced a slight increase in both groups.
Cairo et al. (2017), Italy	RCT	Parallel	Prospective	XCM/CTG; 60 (30/30) Mucograft	6 months	KT:3.1 ± 1.2	STT: 2.1 ± 0.63	KT: 4.3 ± 1.2	STT: 3.0 ± 0.7	KT: 1.1 ± 0.8 STT: 0.9 ± 0.2	CTG proved more effective than XCM in increasing buccal peri‐implant soft tissue thickness. XCM and CTG yielded comparable quantities of KT after a period of 6 months.

Abbreviations: APF: apically positioned flap; BSP: buccal soft tissue profile; CCT: controlled clinical trial; CM: collagen matrix; CTG: connective tissue graft; FGG: free gingival graft; KMT: keratinized mucosa thickness; KMW: keratinized mucosa width; KT: width of keratinized tissue; NR: not reported; RCT: randomized controlled trial; SCTG: subepithelial connective tissue graft; STT: soft tissue thickness; STV: soft tissue volume; VCMX: volume‐stable collagen matrix; XCM: xenogeneic collagen matrix.

#### 3.2.1 Study Design

There were eight randomized controlled trials (RCTs) and one CCT. Sample sizes in these studies ranged from 14 (Schmitt et al. 2021) to 60 (Cairo et al. 2017; Cosyn et al. 2022). The follow‐up duration varied from 6 months (Cairo et al. 2017; Huang et al. 2021; Schmitt et al. 2021; Qui et al. 2023; Ramanauskaite et al. 2023) to 5 years (Thoma et al. 2023). While most studies described the soft tissue augmentation outcome in terms of KMW and STT, two studies (Schmitt et al. 2021; Cosyn et al. 2022) mentioned the outcome measures in terms of STVs.

#### 3.2.2 Effect of Collagen Matrix on Soft Tissue Augmentation

The included studies demonstrated varying results regarding soft tissue augmentation between collagen matrix and autografts (SCTG and FGG). In a 5‐year follow‐up study by Thoma et al. (2023), XCM and SCTG showed similar results in soft tissue augmentation. Similarly, Cairo et al. (2017) demonstrated comparable amounts of keratinized tissue width with XCM and CTG when evaluated after 6 months. XCM, when combined with an apically positioned flap (APF), resulted in similar amounts of KMW augmentation compared to FGG plus APF but with higher shrinkage (Qiu et al. 2023).

On the other hand, some studies showed better results with autografts (control group). FGG with (Solonko et al. 2022) and without APF (Huang et al. 2021; Ramanauskaite et al. 2023) resulted in a wider keratinized tissue band than CM with or without APF. A few studies found a greater increase in STT at implant sites with CTG (Cosyn et al. 2022) and FGG (Huang et al. 2021) than with CMX. Some studies showed no difference between the two groups. Minimal changes in the peri‐implant tissue contour, as well as in STT, were found by Thoma et al. (2020), which slightly increased in both groups (Thoma et al. 2020). Both maintained peri‐implant health and had similar aesthetic outcomes (Huang et al. 2021).

#### 3.2.3 Aesthetics

Improved aesthetics were reported with both XCM and SCTG and clinically negligible contour changes at 5 years after loading (Thoma et al. 2023). Better aesthetic outcomes were reported with XCM plus APF than with FGG plus APF (Qui et al. 2023). However, CMX could result in considerable resorption (Cosyn et al. 2022).

#### 3.2.4 Need for Analgesics

CM reduced the need for analgesics significantly (Ramanauskaite et al. 2023). Similar results were found even when CM was combined with APF (Solonko et al. 2022).

#### 3.2.5 Surgical Time

While Solonko et al. (2022) found similar surgical times, another study (Huang et al. 2021) found XCM reduced operation time.

### 3.3 Risk of Bias Across Studies

The risk of bias in individual RCTs using the Cochrane Collaboration tool (Higgins et al. 2011) and ROBINS‐I for assessing the risk of bias in nonrandomized studies of interventions (Sterne et al. 2016) are presented in Tables 2a and 2b. Among the RCTs, seven studies had a low risk of bias, while one showed a high risk. The nonrandomized trial (Schmitt et al. 2021) showed a moderate risk of bias.

**Table 2a:** Risk of bias of included in randomized trials using Cochrane Collaboration's tool (Higgins et al. 2011).

	Selection bias		Performance bias	Detection bias	Attrition bias	Reporting bias		Risk of bias
Author (year)	Random sequence generation	Allocation concealment	Blinding of participants, personnel	Blinding of outcome assessment	Incomplete outcome data	Selective reporting	Other bias	Summary assessment
Thoma et al. (2023)	+	+	?	+	+	+	+	Low
Qiu et al. (2023)	+	+	+	+	+	+	+	Low
Ramanauskaite et al. (2023)	?	+	?	?	+	+	+	High
Solonko et al. (2022)	+	+	+	+	+	+	+	Low
Cosyn et al. (2022)	+	+	+	+	+	+	+	Low
Huang et al. (2021)	+	+	?	+	+	+	+	Low
Thoma et al. (2020)	+	+	+	+	+	+	+	Low
Cairo et al. (2017)	+	+	?	+	+	+	+	Low

**Table 2b:** ROBINS‐I for assessing risk of bias in nonrandomized studies of interventions (Sterne et al. 2016).

Author (year)	Bias due to confounding	Bias in the selection of participants for the study	Bias in the classification of interventions	Bias due to deviations from intended interventions	Bias due to missing data	Bias in the measurement of outcomes	Bias in the selection of the reported result	Overall risk
Schmitt et al. (2021)	+	?	+	+	+	?	+	Moderate

### 3.4 Meta‐Analysis

Nine studies evaluated the mean differences in KMW and STT/STV, and six of these entered the meta‐analysis. These six studies (Cairo et al. 2017; Thoma et al. 2020; Huang et al. 2021; Solonko et al. 2022; Qiu et al. 2023; Ramanauskaite et al. 2023) reported KMW and STT. The other studies reported STVs (Schmitt et al. 2021; Cosyn et al. 2022) but could not be included in the meta‐analysis since sufficient data were unavailable for Cosyn et al. (2022) at 6 months and Schmitt et al. (2021) at 12 months in terms of STV. Other studies (Thoma et al. 2020; Cosyn et al. 2022) were not included as we have included its 5‐year follow‐up study (Thoma et al. 2023).

**Figure 2:**
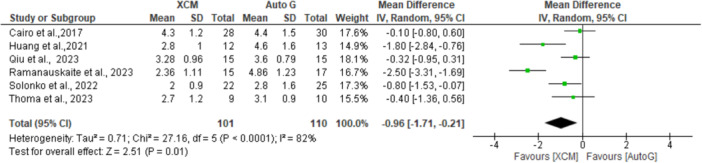
XCM versus autograft for KMW (keratinized mucosa width) at 6‐month follow‐up. The green square represents individual studies effects. The black line represents confidence interval. The diamond represents the overall effect.

Among the studies included in the meta‐analysis, the *I* ^2^ value was high (82%), implying increased heterogeneity across studies; hence, a random‐effects model was chosen (Figure 2). The pooled mean difference was −0.96 (−1.71 to −0.21) between XCM and autograft groups, showing an increased KMW in the autograft group compared to XCM (*p* < 0.05).

**Figure 3:**
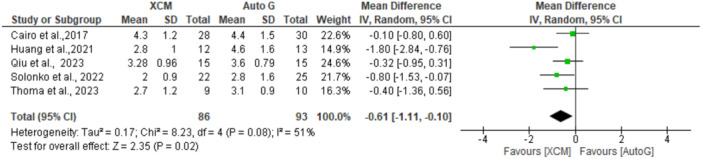
Sensitivity analysis: XCM versus autograft for KMW (keratinized mucosa width) at 6‐month follow‐up. The green square represents individual studies effects. The black line represents confidence interval. The diamond represents the overall effect.

A sensitivity analysis was conducted by excluding the article with a high risk of bias (Ramanauskaite et al. 2023), resulting in decreased heterogeneity across studies (*I* ^2^ = 51%). Hence, a random‐effects model was chosen (Figure 3). The pooled mean difference was −0.61 (−1.11 to −0.10) between XCM and autograft groups, implying an increased KMW in the autograft group compared to XCM (*p* < 0.05).

**Figure 4:**
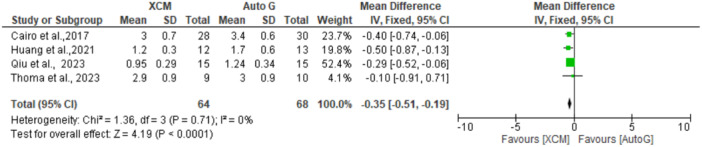
XCM versus autograft for STT (soft tissue thickness) at 6‐month follow‐up. The green square represents individual studies effects. The black line represents confidence interval. The diamond represents the overall effect.

When STT at the 6‐month follow‐up was assessed for all five studies (Figure 4), the *I* ^2^ value was low (0%). This meant less heterogeneity across studies; hence, a fixed‐effects model was chosen. The pooled mean difference was −0.35 (−0.51 to −0.19) between XCM and autograft groups, implying increased STT in the autograft group compared to XCM (*p* < 0.05).

**Figure 5:**
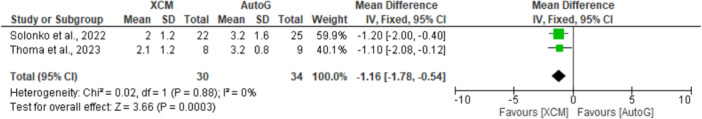
XCM versus autograft for KMW at 12‐month follow‐up. The green square represents individual studies effects. The black line represents confidence interval. The diamond represents the overall effect.

When the KMW of all studies included were assessed at the 12‐month follow‐up (Figure 5), the *I* ^2^ value was low (0%), implying less heterogeneity across studies; hence, a fixed‐effects model was chosen. The pooled mean difference was −1.16 (−1.78 to −0.54) between XCM and autograft groups, showing an increased KMW in the autograft group compared to XCM (*p* < 0.05).

## 4 Discussion

The objective of the present systematic review and meta‐analysis, framed in alignment with the PICO question, addresses the efficacy of XCMs in improving two specific variables: KMW and STV, including STT. This review compares XCM (Mucograft) with other collagen matrices and only looks at cases without bone grafts. Nine studies met the inclusion criteria and were considered for the review. However, only six studies were suitable for the meta‐analysis because three were excluded due to differences in measurement units. The excluded studies, namely those by Schmitt et al. (2021) and Cosyn et al. (2022), using different measurement units employing cubic millimeters (mm^3^) to represent STV. This differed from the measurement units used in the other studies included in the meta‐analysis, making it challenging to combine the data for a unified statistical analysis. Specifically, Thoma et al. (2020) and Cosyn et al. (2022) lacked available measurements for inclusion in the meta‐analysis. Concurrently, Schmitt et al. (2021) presented no data regarding STV at the 12‐month measurement point.

In the scientific endeavor to elucidate the comparative efficacy of XCM and autogenous grafts in soft tissue augmentation around dental implants, the present systematic review adopts a nuanced approach. This study stands out by only focusing on studies that do not use bone grafts. While this limits the amount of data available, it improves the depth and specificity of the analysis of soft tissue outcomes. So far, no other reviews have focused on this area, making this study the first of its kind in this academic field.

The meta‐analysis reveals a discernible pattern: autogenous grafts manifest superior efficacy in KMW and STV, including STT when contrasted with XCM. This corroborative evidence aligns with previous meta‐analyses that have postulated similar outcomes. Noteworthy examples include studies by Thoma et al. (2014), Moraschini et al. (2020), and Valles et al. (2022), which have mainly highlighted the increased STV associated with autogenous grafts vis‐à‐vis XCMs. However, the literature on KMW demonstrates more significant heterogeneity in results. For instance, a recent systematic review by Montero et al. (2022) demonstrated that autogenous grafts, specifically free gingival grafts, are substantially more efficacious in augmenting KMW than their soft tissue substitute counterparts. Conversely, a RCT published by Cairo et al. (2017) contends for parity between XCM and CTGs regarding final keratinized tissue amounts after 6 months. The idea of similarity is supported by a study by Qiu et al. (2023), even though an apically positioned flap was used in both treatment methods.

Including recent studies by Huang et al. (2021) and Ramanauskaite et al. (2023) further enriches the discourse on the relative efficacies of autogenous grafts and XCM in soft tissue augmentation around dental implants. Both of these studies emphasize the superiority of autogenous grafts, specifically free gingival grafts, over XCM in augmenting KMW. Interestingly, these findings contrast with the study by Cairo et al. (2017), where CTGs, rather than free gingival grafts, were used, resulting in similar KMW outcomes compared to XCM. This suggests that the type of autogenous graft used significantly influences the outcomes, a point that future research should explore.

Regarding STT, most of the reviewed literature, including studies by Cairo et al. (2017), Qiu et al. (2023), Cosyn et al. (2022), and Huang et al. (2021), corroborates the superior performance of autogenous grafts, particularly at the 6‐ and 12‐month follow‐up periods. However, the study by Thoma et al. (2020) with a 3‐year follow‐up challenges this narrative. According to this study, soft tissue grafts and a specific type of XCM resulted in a comparable increase in STT. A subsequent 2‐year follow‐up by Thoma et al. (2023) further supports this finding. Thus, while autogenous grafts may exhibit superior outcomes in the short term, XCMs may offer comparable results in the long term for specific metrics, such as STT. Systematic reviews by Gargallo‐Albiol et al. (2019) and Cairo et al. (2019) also note the superior efficacy of CTG over XCM in STT, albeit with marginal differences ranging from 0.19 to 0.30 mm. Though these differences might appear minor, they could be clinically significant and worth considering in treatment planning and outcome evaluations.

The data from Schmitt et al. (2021) that compared CTGs with porcine acellular dermal matrix (PADM), specifically mucoderm, found CTG more effective in both STT and STV increase after 6 months. This corroborates the general trend in the literature favoring autogenous grafts, particularly CTG. Similarly, the Cosyn et al. (2022) study reaffirmed the superiority of CTG over the XCM in improving STT, strengthening the case for CTG being the “gold standard.” Interestingly, the review revealed that the choice of material for soft tissue substitutes varies considerably across studies, from bilayered collagen matrices to volume‐stable collagen matrix (VCMX) and PADM. This diversity in choices echoes previous literature; while bilayered collagen matrices have been a common choice (Sanz et al. 2009; Lorenzo et al. 2012), other types of materials like PADM have started to appear in more recent studies (Zafiropoulos et al. 2016; Papi and Pompa 2018; Schmitt et al. 2021). The study by Happe et al. (2022) is intriguing as it brings in the dimension of bone grafting in combination with soft tissue augmentation. The similar outcomes in terms of horizontal change for both the CTG and PADM groups suggest that, when combined with bone grafts, the type of soft tissue augmentation material might not drastically affect the outcome, at least in ridge dimensions.

Our review highlights an exciting trend in dental research—the advent of newer, cross‐linked collagen matrices like VCMX (Fibrogide), designed for better volume maintenance. Recent multicenter studies like that of Hammerle et al. (2023) exhibit encouraging data, showing comparable buccal volume gains to the established SCTG, even if STT favored the latter. These hold promise for XCM's efficacy and add a new layer of complexity to the existing literature.

Equally exciting is the evolving dialogue around aesthetic outcomes. While earlier studies had already shown the promise of matrices like PADM in aesthetics, newer research by Qiu et al. (2023) and Manfredini et al. (2023) indicates superior aesthetic outcomes with bilayered collagen matrices. These findings are compelling because they demonstrate long‐term stability in aesthetic results, a crucial factor often underestimated in previous studies. Long‐term observational studies like those by Thoma et al. (2023) and Happe et al. (2022) offer valuable insights into the sustainability of both aesthetic and functional outcomes. Such long‐term data, though limited, can serve as robust evidence for clinicians when making treatment choices.

While the advancements in collagen matrices are promising, graft shrinkage remains a universal challenge, affecting both SCTGs and collagen matrices. Studies like Cosyn et al. (2022) and Eeckhout et al. (2020) quantify this phenomenon, providing essential data for clinical decision‐making. It is noteworthy that Ramanauskaite et al. (2023) showed comparable shrinkage rates between FGG and Mucograft from 3 to 6 months, a finding that requires further exploration. Data from Fischer et al. (2019) adds an intriguing layer to the tissue shrinkage discourse by illustrating that volume loss may stabilize after an initial period, suggesting that volume maintenance could be time‐dependent. These insights necessitate more longitudinal studies to understand tissue shrinkage and regeneration dynamics better.

## 5 Limitations and Strengths of This Review

A noteworthy limitation of this review is the predominance of studies that focus on XCM (mucograft) as the collagen matrix of choice, leaving other promising matrices like PADM and VCMX underrepresented. In our review, only one study (Schmitt et al. 2021) utilized PADM and three (Thoma et al. 2020, 2023; Cosyn et al. 2022) employed VCMX, making it difficult to compare the efficacy of these different matrices comprehensively. However, emerging literature suggests a shift toward including a broader range of collagen matrices, especially cross‐linked types, as evidenced by recent studies (Cosyn et al. 2022; Hammerle et al. 2023).

Another constraint is the focus of many included studies on immediate implants, often incorporating bone grafts to fill the extraction socket, particularly the vestibular gap. This trend narrows the available pool of studies that deal solely with soft tissue augmentation, thus limiting our ability to directly measure the effectiveness of these procedures in line with the objective of this review. The inclusion of short‐term follow‐up studies further complicates this. Only two studies by Thoma et al. (2020, 2023) offered long‐term data, making the evaluation of graft stability more challenging. It should also be noted that two studies lacking 6‐ and 12‐month data were excluded from the meta‐analysis, contributing to increased heterogeneity and reducing the number of available studies for evaluating efficacy.

Additionally, varying methodologies across studies, such as conventional endodontic files versus digital casts and CBCT, introduce a more significant margin of error in data collection (Ashurko et al. 2023). Despite these limitations, this review has several strengths. The data extraction and screening process was rigorous, enhancing the systematic review and meta‐analysis quality. Unlike previous reviews that combined studies of soft tissue augmentation with bone grafting, this review focused exclusively on soft tissue outcomes. Most of the included studies were randomized CCTs, with only one being a CCT, thus significantly reducing the risk of bias. Moreover, the review is timely, capturing several high‐impact RCTs published in the last 2 years that explore collagen matrices beyond mucograft, thus adding a newer dimension to the existing body of literature (Cosyn et al. 2021, 2022; Happe et al. 2022; Hammerle et al. 2023).

## 6 Recommendations for Future Studies

Future research should prioritize longer follow‐up periods to gauge the long‐term stability of grafts, a vital consideration for patient satisfaction and treatment efficacy. Additionally, the growing body of literature around new collagen matrices like VCMX suggests more RCTs incorporating a broader range of collagen matrices, thus providing a more robust data set. Another vital research avenue is the design of studies that exclusively focuses on soft tissue augmentation, avoiding the inclusion of bone grafts, which can confound measurement outcomes. Instrumentation for measuring STV could also benefit from technological advancements. The traditional endodontic file has limitations in accuracy; hence, newer methods involving digital workflows, intraoral scanners, and CBCT should be utilized for greater data integrity. Furthermore, future studies should aim for greater standardization to control confounding variables such as smoking status, gingival phenotype, implant type, and surgical methodology. Standardizing the timing of graft placement could also bring more uniformity to the results.

## 7 Conclusions

Collagen matrices, compared to autogenous grafts, were less effective in increasing both keratinized tissue and STV, including thickness. Despite this, no significant differences were noted in aesthetic outcomes between the two types of grafts. There is a pressing need for more elaborate randomized controlled trials with extended follow‐ups and larger sample sizes to make more nuanced comparisons between different collagen matrices. Moreover, additional studies focusing solely on soft tissue augmentation around implants, particularly in the absence of bone grafts, are essential for a more comprehensive understanding of the topic. This review aims to elucidate the current landscape of using collagen matrices for soft tissue augmentation in dental implants, highlighting strengths and areas that require further investigation to inform clinical practice better and improve patient outcomes.

## Author Contributions

Study concept and design: S.D. and B.J. Analysis and interpretation of data: S.A., S.D., and B.J. Drafting of the manuscript: S.A., S.D., and B.J. Critical revision of the article: S.A. Approval of the article: S.A. Statistical analysis: B.J. Data collection: B.J. and S.D. Data analysis: B.J. and S.D.

## Ethics Statement

The protocol is registered in the International Prospective Register of Systematic Reviews (PROSPERO) with ID No. CRD42023455643.

## Consent

The authors have nothing to report.

## Conflicts of Interest

The authors declare no conflicts of interest.

## Data Availability

The data sets used and/or analyzed during the current study and the data that support the findings of this study are available from the corresponding author upon reasonable request.
